# Current Understanding of the Neural Mechanisms of Dissociation in Borderline Personality Disorder

**DOI:** 10.1007/s40473-018-0146-9

**Published:** 2018-02-12

**Authors:** Annegret Krause-Utz, Bernet Elzinga

**Affiliations:** 1 Institute of Psychology, Leiden University, Leiden, The Netherlands; 2 Leiden Institute for Brain and Cognition (LIBC), Leiden, The Netherlands

**Keywords:** Borderline personality disorder, Depersonalization, Dissociation, Traumatic stress, Neuroimaging

## Abstract

**Purpose of Review:**

In this article, we aim to give an overview over recent neuroimaging research on dissociation in borderline personality disorder (BPD). Stress-related dissociation is highly prevalent in BPD, while so far only little is known about its neural underpinnings.

**Recent Findings:**

Based on research in depersonalization and the dissociative subtype of posttraumatic stress disorder, it has been proposed that dissociation involves alterations in a cortico-limbic network. In BPD, neuroimaging research explicitly focusing on dissociation is still scarce.

**Summary:**

Functional neuroimaging studies have provided preliminary evidence for an altered recruitment and interplay of fronto-limbic regions (amygdala, anterior cingulate, inferior frontal gyrus, medial and dorsolateral prefrontal cortices) and temporoparietal areas (superior temporal gyrus, inferior parietal lobule, fusiform gyrus), which may underlie disrupted affective-cognitive processing during dissociation in BPD. More neuroimaging research with larger samples, clinical control groups, and repeated measurements is needed to deepen the understanding of dissociation in BPD.

## Introduction

Stress-related dissociative states, including depersonalization, derealization, analgesia, and emotional numbing, are a clinical hallmark of borderline personality disorder (BPD), occurring in about 75–80% of patients [1–6]. In the current version of the *Diagnostic and Statistical Manual of Mental Disorders* (DSM5), transient “dissociative states under stress” are listed among the diagnostic features of BPD [7]. Such states usually last for minutes or hours [8–10] and are often linked to stress-driven rapid shifts in self-image [10]. The intensity, frequency, and strength of self-reported dissociative states in BPD seem to linearly increase with the level of subjective distress [11]. Furthermore, there is evidence for a detrimental effect of dissociation on emotional learning and memory [12–15], which may partly explain why dissociative symptoms can contribute to poor therapy outcome in patients with BPD [16–19]. So far, very little is known about the neural mechanisms of disturbed information processing during dissociation: Only few neuroimaging studies in BPD explicitly focused on dissociation, compared to the growing body of research in other trauma-related and dissociative disorders (e.g., dissociative subtype of posttraumatic stress disorder).

In the following, we introduce current conceptualizations of dissociation, based on more substantial neuroimaging research that has been conducted in patients with depersonalization, the dissociative subtype of posttraumatic stress disorder, and dissociative identity disorder (a comprehensive review of this literature is beyond the scope of this article; for further reviews see, e.g., [20–25]). Based on this literature, we then describe and discuss recent neuroimaging research on dissociation in BPD (focusing on functional neuroimaging studies) together with its implications, methodological limitations, and suggestions for future research.

### Definitions and Etiological Models of Dissociation

Dissociation has been defined as “disruption of and/or discontinuity in the normal, subjective integration of one or more aspects of psychological functioning, including – but not limited to – memory, identity, consciousness, perception, and motor control” [24] (p. 826). The broad variety of symptoms can be classified into the following three categories: (1) a loss of continuity in subjective experience, accompanied by involuntary and unwanted intrusions into awareness or behavior (e.g., dissociative flashbacks); (2) an inability to access information or control mental functions that are normally amenable to such control or access (e.g., dissociative amnesia); and (3) a sense of experiential disconnectedness, including distorted perceptions about the self or the environment (e.g., depersonalization, derealization) [26]. Acute dissociative states (*state dissociation*) are usually distinguished from a more general tendency to experience dissociative symptoms (*trait dissociation*), while both may go hand in hand. Of note, dissociative experiences are not necessarily pathological but, to a lesser extent, are also present in non-clinical healthy populations [27, 28]. The present article focuses on pathological dissociation, as highly prevalent in BPD, PTSD, and dissociative disorders [9, 10, 29–31].

#### Etiological Models

Interacting with genetic, neurobiological, and cognitive dispositions, traumatic stress can play an important role in the development of pathological dissociation [32–44]. A history of trauma is neither necessary nor sufficient for the development of dissociation and/or BPD. Yet, dissociation in BPD has been closely linked to chronic interpersonal trauma, such as severe childhood abuse and neglect [6, 25, 45, 46]. *Peritraumatic dissociation* immediately during the traumatic event [34] has been partly conceptualized analogous to freezing responses in animals. The proximity of threat may first elicit an orienting response (*fight or flight* response; [47]), associated with an increase in sympathetic nervous system function (e.g., heart rate, blood pressure, release of stress hormones), to prepare the organism for an active defense mechanism. If the organism cannot escape, it may more likely engage in a passive defense mode, accompanied by tonic immobility (freezing), an increase in parasympathic activity and a shutting-down of the arousal system [33, 38, 48, 49]. If chances of winning a fight or escaping are low or not given, passive defense responses such as tonic immobility may reduce the risk of being detected or more severely injured [48, 50]. In general, direct translations from animal studies to humans are not possible due to conceptual and methodological differences [49]. Yet, dissociative responses, such as tonic immobility, in humans might be an evolutionary-based regulatory process, which may enhance survival or the coping with extreme threat [22, 25, 26, 38, 44, 51]. The traumatic event may be perceived as a film-like scene from a wider distance. Salient sensory information may be processed in a distorted way and perception of time may be substantially altered. Emotional numbing and depersonalization may help create an inner distance from overwhelming and horrifying experiences that cannot be integrated into existing views of the world, self, and others. Such distortions in time, thought, body (e.g., out of body experiences, such as the sense of floating above one’s body), and emotions may later re-occur as distinctive “trauma-related states of consciousness” (TRASC) [52]. It has been shown that individuals who experienced peritraumatic dissociation are more likely to dissociate in other stressful life situations later on in life [34, 52–54]. Thereby, dissociation can interfere with the development of identity [24, 38, 55] and lead to a compartmentalization (fragmentation) of memories [56]: Salient characteristics of the stressful event are encoded and stored as separate elements, which may later re-occur as unwanted implicit flashback memories [51, 57–60], increasing the risk of posttraumatic stress symptoms [34, 54, 61, 62]. Several studies provided evidence for a disruptive effect of dissociation on neuropsychological functioning [13, 51, 57, 63], although this relationship is not yet completely understood and cannot be generalized across different (clinical) groups [64–66].

### Neurobiological Conceptualizations of Dissociation

While the precise neural mechanisms of dissociation remain elusive, neuroimaging research in depersonalization disorder, dissociative subtype (D-PTSD), and dissociative identity disorder has provided important insights into brain networks possibly implicated in dissociation. In the following, current neurobiological conceptualizations stemming from this research are briefly introduced.

#### Cortico-Limbic Disconnection Model: Research in Depersonalization Disorder

Already in 1998, Sierra and Berrios proposed that a “disconnection” of cortico-limbic brain regions underlies symptoms of depersonalization, such as numbing, analgesia, hypervigilance, and emptiness of thoughts [67]. In their cortico-limbic disconnection model, the authors propose that increased activity in medial and dorsolateral prefrontal cortices (areas implicated in cognitive control and arousal modulation) both directly and indirectly via the anterior cingulate cortex (ACC) [68–70] dampens activity in the amygdala, which is crucial to the initiation of stress and fear responses [71, 72]. In addition to the aforementioned cortico-limbic regions, the posterior cingulate, hippocampus, superior temporal gyrus, precuneus, temporoparietal junction, inferior parietal lobe, angular gyrus, frontopolar cortex, and medial prefrontal cortex (mPFC) have been implicated in states of depersonalization [73–75]. These areas are parts of the default mode network, which has been implicated in inward-directed processes, such as autobiographical memory retrieval, daydreaming, rumination, pain processing, and mentalizing [76–81]. Alterations in the abovementioned regions may underlie changes in self-perception, such as “feeling unreal” [67, 82] and altered interoceptive awareness during depersonalization [68, 74, 75, 83] [84–86].

#### Emotion Overmodulation: Research in the Dissociative Subtype of PTSD and in DID

Further evidence for alterations in cortico-limbic and default mode network regions stems from research in PTSD patients with the D-PTSD, which has recently been introduced in DSM5 [7]. As proposed by Lanius and colleagues [22] (p. 640), dissociation may involve an increase in self-control and arousal modulation (“emotion overmodulation”), associated with increased activation in frontal regions (dorsal/rostral ACC, mPFC) and dampened limbic activity in the amygdala and insula. Patients predominantly showing hyperarousal and re-experiencing symptoms (emotion under-modulation) are thought to exhibit the reverse pattern: limbic hyperactivity and diminished recruitment of the ACC and mPFC. Central to this model were observations from a script-driven imagery study, in which PTSD patients were exposed to autobiographical narratives of traumatic events [87, 88]. The majority of patients reported pronounced re-experiencing symptoms (traumatic flashbacks, intense feelings of shame, disgust, guilt, hopelessness, etc.), associated with an increase in heart rate. In contrast, patients with the dissociative subtype demonstrated increased activity in areas implicated in emotion regulation, arousal modulation, and sensory filtering (e.g., medial frontal gyrus, anterior cingulate, middle temporal gyri, precuneus, occipital areas, and inferior frontal gyrus) [87]. Patients with the dissociative subtype exhibited a stronger coupling of the ventrolateral thalamus with right insula, middle frontal gyrus, superior temporal gyrus, cuneus, and left parietal lobe (regions implicated in emotion regulation), while connectivity between the thalamus and right parahippocampal gyrus and superior occipital gyrus (areas implicated in sensory and emotion processing) was diminished [88]. The thalamus functions as a sensory gate or filter, receiving direct input from both subcortical limbic regions and frontal areas, and may therefore play an important role in dissociative-like states of altered consciousness [21, 89, 90]. More specifically, the thalamus may either facilitate or impede the flow of information to cortico-limbic structures [21] and thereby contribute to defensive regulatory processes [91, 92].

Interestingly, patients with the dissociative subtype exhibited enhanced activity in the ventral PFC, suggesting increased emotion downregulation, when threatening (fearful versus neutral) facial expressions were presented on a conscious level—but not when threatening stimuli were presented non-consciously. In the latter condition, these patients showed increased activity in the amygdala, insula, and thalamus, suggesting that regulatory processes during dissociation may be partly explained by conscious top-down processes, which might not work on a non-conscious level [91].

Further evidence for altered fronto-limbic interactions, in terms of a stronger coupling of the amygdala with fronto-parietal regions, stems from resting-state functional magnetic resonance imaging (RS-fMRI) studies investigating connectivity patterns in the absence of experimental stimulation [93]. Nicholson and colleagues (2017) used dynamic causal modeling (DCM) to more directly investigate directed (effective) connectivity between functional nodes of fear and emotion regulation circuits [94]. In PTSD patients with the dissociative subtype, a predominate pattern of top-down connectivity from the ventromedial PFC (vmPFC) to the amygdala and periaqueductal gray (PAG) and from the amygdala to the PAG was found. In contrast, PTSD patients without the dissociative subtype predominately showed bottom-up connectivity from the amygdala to the vmPFC and from the PAG to the vmPFC and amygdala. These differences in resting-state connectivity in brain circuits associated with fear processing [94] may translate into symptoms of emotion over—versus under—modulation in PTSD patients with versus without the dissociative subtype [95, 96]. In this context, the PAG may play an important role in passive defense responses during dissociation [97].

Higher depersonalization/derealization severity further predicted decreased connectivity between the perigenual ACC and ventromedial PFC as well as altered synchrony between the default mode network and the central executive network [98]. Extending the abovementioned finding, these results point to an altered synchrony, i.e., switching between large-scale networks implicated in rest and executive control in the dissociative subtype of PTSD.

Interestingly, patients with dissociative identity disorder (DID) demonstrated distinct neural response patterns, depending on the presence or absence of dissociation, which highly resemble observations in PTSD: While being in a “hyper-aroused state” or “traumatic identity state” with voluntary access to traumatic memories, DID patients showed elevated cardiovascular responses (heart rate, blood pressure) and stronger amygdala and insula activity, along with lower activity in cingulate gyrus, parietal cortex, and para-hippocampus. The opposite response pattern was observed when patients were in their “normal dissociative identity state,” characterized by dissociative amnesia [99, 100]. Studies including clinical control groups are needed to clarify whether altered activity patterns in fronto-limbic areas described above are specific to the dissociative subtype of PTSD or a trans-diagnostic phenomenon of emotion overmodulation, extending to other stress-related disorders including DID and BPD.

### Neuroimaging Research on Dissociation in BPD

Compared to research in D-PTSD and dissociative disorders, studies explicitly focusing on dissociation in BPD are still relatively scarce. Most evidence stems from studies that examined correlations between neuroimaging findings and clinical measures of dissociation, without explicitly and/or exclusively focusing on dissociation or inducing this symptom [101–103] [104–116]. To our knowledge, so far, only three studies used script-driven imagery to more directly investigate the effect of experimentally induced dissociation on neural processing in BPD. Findings of functional neuroimaging studies are described in more detail below.

#### Resting-State Neuroimaging Studies

Several studies in BPD examined links between self-reported dissociation and findings of altered baseline glucose metabolism and cerebral blood flow during rest. Several studies using positron emission tomography (PET) [104], single-photon emission computed tomography (SPECT) [105], or continuous arterial spin labeling [106] did not report significant links between altered baseline metabolism/activity and dissociation, while self-reported dissociation predicted changes in resting-state functional connectivity (RSFC) in two fMRI studies [107, 108]. Wolf and colleagues (2011) found positive correlations between scores on the Dissociation Stress Scale (DSS) and RSFC in the insula and precuneus. Krause-Utz and colleagues (2014) investigated whether scores on the Dissociative Experiences Scale (DES) [109] predicted RSFC of the bilateral amygdala and ACC in BPD. Higher trait dissociation positively predicted amygdala RSFC with the dorsolateral prefrontal cortex (dlPFC) and negatively predicted left amygdala RSFC with a cluster in the occipital lobe (cuneus, intracalcarine cortex, and fusiform gyrus). More resting-state fMRI studies with larger sample sizes and clinical control groups are needed to understand the precise mechanisms underlying the abovementioned RSFC patterns in BPD. Future studies in BPD may acquire resting-state scans before and after dissociation induction or therapeutic interventions aimed at a reduction of dissociation to gain more insight into the impact of dissociation on RSFC in large-scale brain networks.

#### Task-Related fMRI Studies

Some studies in BPD reported significant correlations between clinical measures of dissociation and changes in blood-oxygen-level-dependent (BOLD) responses during the presentation of aversive images [110–112]. Hazlett and colleagues (2012) found negative correlations between self-reported dissociation and amygdala reactivity during a habituation task with repeatedly presented (versus novel) unpleasant pictures in BPD patients and patients with schizotypal personality disorder. Overall, BPD patients showed increased amygdala reactivity to repeatedly presented (versus novel) emotional pictures and a prolonged return to baseline of amygdala compared to healthy controls. In line with this, Krause-Utz and colleagues (2012) found significant associations between self-reported dissociation and amygdala reactivity to negative pictures, presented as distractors in the context of a working memory task, in BPD. The Emotional Working Memory Task (EWMT) used in this study is a modified version of a Sternberg item-recognition task, in which participants are instructed to memorize task-relevant information (a set of letters) over a short delay interval. Afterwards, a probe (another set of letters) is presented and participants are instructed to indicate whether one of these letters was part of the previous set (memoranda) or not by pressing a “yes” or “no” button. In half of the trials, a target is present, while in the other trials, the target is absent. During the delay interval, either no distractors (only a fixation cross) or distracting neutral versus negative pictures of interpersonal scenes are presented. Negative pictures contain scenes of interpersonal violence, such as sexual or physical assault, a beaten child, or a physically mutilated body. Participants are instructed to ignore these distractors and to respond as fast and accurately as possible to the probes, i.e., to voluntarily inhibit emotion processing in favor of cognitive processing. During presentation of neutral and negative interpersonal scenes, BPD patients showed significantly longer reaction times and significantly increased activity in the amygdala (among other regions), suggesting higher emotional distractibility than healthy controls. At the same time, self-reported dissociation was negatively correlated to amygdala activity in BPD.

A re-analysis of this data set showed that self-reported state dissociation further positively predicted functional connectivity of the amygdala with right thalamus, right ACC, left insula, and left precentral gyrus during negative distractors [112]. Altered interaction between these regions may underlie altered processing of disturbing emotional information in the context of a cognitive task in BPD.

In another study, higher trait dissociation (scores on the DES) predicted a stronger signal decrease of the default mode network in response to painful heat stimulation in BPD patients with current self-injurious behavior [113]. As previously mentioned, the default mode network plays an important role in self-referential processing and pain processing. Alterations in this network might underlie altered processing of pain, e.g., as being less self-relevant or aversive, in patients with frequent dissociative experiences. Since in other studies no significant correlations between dissociation and limbic reactivity during painful heat stimulation [114, 115] or emotional words [116, 117] were found, future research is needed to investigate whether different kinds of experimental material and subsamples may have contributed to these discrepancies.

#### Script-Driven Imagery Studies

To our knowledge, so far, three fMRI studies used script-driven imagery to experimentally induce dissociation in individuals with BPD [117–119]. Script-driven imagery is a well-established paradigm, aimed at provoking specific experiences through a recollection of autobiographical memories. Together with the experimenter, each participant creates a personalized narrative of an autobiographical situation, e.g., a situation involving marked dissociative experiences (versus an emotionally neutral script). The script is usually recorded on an audio tape by the experimenter and later re-presented in an experimental setting, e.g., during fMRI. Participants are instructed to recall the specific situation, described in the script, as vividly as possible. Consistent across different studies, script-driven imagery successfully induced dissociation, in terms of self-reported dissociative symptoms (on the DSS scale) in previous research [117–119]. In a pilot study by Ludaescher and colleagues (2010), self-reported state dissociation, pain sensitivity, and changes in BOLD responses were assessed in 15 BPD patients exposed to either a dissociation script or a neutral script. While being exposed to the dissociation script, patients showed increased activation in the left inferior frontal gyrus. Higher self-reported dissociative symptoms predicted higher activation in the left superior frontal gyrus and lower activation in the middle and inferior temporal gyrus. BPD patients with comorbid PTSD (*n* = 10) additionally showed increased activation in the left cingulate gyrus. In this subgroup, self-reported dissociation positively predicted activity in the bilateral insula, while negatively predicting activity in the right parahippocampal gyrus. Findings of this study provided first evidence for increased frontal activity and diminished temporo-limbic activity during acute dissociation in BPD, especially in a subgroup of patients with comorbid PTSD. However, the sample size in this study was relatively small and no control group was included.

Winter and colleagues (2015) combined script-driven imagery with the Emotional Stroop Task (EST) and subsequent memory tasks to investigate the impact of dissociation induction on the cognitive inhibition of emotional material in BPD. In the EST, either neutral or emotional words are presented in different colors. Participants are instructed to name the color of each word as fast and accurately as possible while ignoring its content. Before they performed the EST, 19 female BPD patients were exposed to a dissociation script (BPDd), while 18 BPD patients and 19 healthy controls were exposed to neutral scripts (BPDn). Patients who underwent dissociation induction showed more errors and slower reaction times than the other groups, together with longer reaction times in response to negative versus neutral words and impaired recall and recognition of EST words during a subsequent memory task compared to BPDn. On a neural level, patients who underwent dissociation induction showed increased activity in the left inferior frontal gyrus and dlPFC for negative versus neutral words and overall lower activity in the fusiform gyrus and inferior parietal and temporal cortices. Altered activity in brain regions implicated in interference inhibition [120] may underlie the disruptive effect of dissociation on cognitive performance in BPD.

Krause-Utz and colleagues (2017) combined script-driven imagery with the EWMT, described above, to investigate the effect of dissociation on working memory performance during emotional distraction. As mentioned above, a previous study using this task revealed negative associations between state dissociation and amygdala activity during emotional distraction in BPD [111]. When performing the EWMT after dissociation induction, BPD patients showed significantly more errors and misses across all conditions compared to healthy controls and BPD patients exposed to a neutral script. BPD patients who underwent dissociation induction further showed an overall deactivation in the bilateral amygdala and lower activity in left cuneus, lingual gyrus, and posterior cingulate compared to patients with a neutral script. During emotional distraction, a stronger positive coupling of the amygdala with right middle/superior temporal gyrus and left inferior parietal lobule along with negative functional connectivity between amygdala and fusiform gyrus was found in patients after dissociation induction. These findings seem to provide further evidence for a disruptive effect of dissociation on affective-cognitive processing in BPD.

### Overall Discussion

The aim of this article was to give an overview over neuroimaging studies on dissociation in BPD. Research in dissociative disorders and D-PTSD provided evidence for alterations in (pre)frontal, subcortical limbic, and tempo-parietal areas (parts of the default mode network). Extending previous observations in depersonalization and D-PTSD [82, 88, 98], dissociative states in BPD have been associated with reduced activation in the inferior and superior temporal gyrus [117, 121] and a stronger positive coupling of this area with the amygdala during emotional distraction [118]. In addition, larger gray matter volumes in the middle and superior temporal gyrus were linked to higher trait dissociation in these patients [103]. Findings in BPD further point to a diminished responsiveness or deactivation of the amygdala during the presentation of aversive (trauma-related) pictures [110, 111, 118], while it remains unclear whether this observation is related to certain tasks or sample characteristics (e.g., specific experimental stimuli, trauma history, medication). The amygdala is crucial to emotion process and the initiation of stress responses [71] and has been critically implicated in neurobiological models, conceptualizing dissociation as self-regulatory strategy to cope with overwhelming emotions [22, 67]. In line with this, altered interactions of the amygdala with brain regions implicated in arousal modulation, body movements, and the filtration of sensory information (ACC, insula, dlPFC, precentral gyrus, fusiform gyrus, inferior parietal lobule, and thalamus) were observed in BPD patients with acute dissociation. A reduced coupling of the amygdala with occipital areas (fusiform gyrus), observed both during resting-state [108] and emotional distraction [112], may translate into altered gating and reduced processing of sensory input. Increased positive amygdala functional connectivity with the dlPFC during rest [108] and with the ACC, insula, and inferior parietal lobule during emotional distraction [112] may reflect increased attempts of arousal modulation, as suggested by research in the dissociative subtype of PTSD [22]. Stress-related dissociation in BPD may be a form of emotion modulation, especially in patients with severe childhood trauma, while so far there is not enough known about neural mechanisms which may underlie these processes [25, 29]. Studying the modulating effect of dissociation on amygdala reactivity and connectivity in BPD may have important implications for neurobiological conceptualizations of the disorder, as amygdala hyperreactivity is currently considered a general key feature of the disorder, despite conflicting findings [122, 123].

Three fMRI studies in BPD consistently observed increased activity in the left inferior frontal gyrus after dissociation induction [117–119], possibly underlying altered suppression of impulses during emotional interference [120]. In the study by Winter and colleagues (2015), BPD patients performing an EST after dissociation induction further showed increased activity in the dlPFC, which plays an important role in emotion downregulation [87, 88]. Alterations in the abovementioned areas may underlie altered information processing during dissociation in BPD. However, the precise neural mechanisms of dissociation need yet to be elucidated and findings need to be interpreted in the light of certain limitations, as discussed below.

#### Limitations

Differences in neuroimaging techniques and sample characteristics (gender, comorbidities, medication status, etc.) hinder the comparison and interpretation of findings. Only few studies included clinical control groups and comorbidity between BPD and other disorders characterized by high trait dissociation is considerably high [9, 10, 29, 31]. Since these disorders may share not only an overlap of symptoms but also etiological factors (e.g., trauma history), more research with clinical control groups and traumatized individuals, who did not develop a disorder, is needed to disentangle disorder-specific from trans-diagnostic effects. Moreover, it remains unclear whether stress-related dissociation in BPD may predominately involve bottom-up or top-down regulatory processes. Studying directed (effective) connectivity between functional nodes of emotion regulation circuits, e.g., using dynamic causal modeling (DCM), may help gain more insight into these processes [94]. Moreover, longitudinal studies may help to clarify whether altered neural response patterns, observed in previous research, are a predisposition for or the result of frequent dissociative experiences. Cross-sectional correlational designs do not allow causal conclusions, e.g., it is also conceivable that patients who are more prone to dissociation show reduced awareness of emotions (alexithymia), which contributes to the observed correlations. A combination of different neuroimaging techniques, such as functional and structural MRI, in future research may help to obtain a more global picture of neural alterations associated with dissociation [124]. The diversity of neuroimaging techniques (e.g., region of interest analyses and seed-based correlations versus data-driven clustering methods) makes it difficult to compare findings across studies, as each technique entails certain advantages and limitations (see [125]). Given the general concerns about robustness and reproducibility of neuroimaging findings, large-scale meta-analyses, including original data sets and software, are needed to tease apart robust results from false-positive findings, especially for findings which are not corrected for multiple comparisons [125]. Finally, sample sizes of most studies are relatively small and studies with larger data sets are needed to replicate or extend existing findings.

#### Implications

Pathological dissociation is a complex heterogeneous phenomenon comprising various symptoms [26–28, 42, 52]. A more precise differentiation between these symptoms in future fMRI research may help to clarify whether neural alterations are specifically related to distinct dissociative features, such as distortion in time, thought, body, and emotion [52]. Likewise, an extended and more precise neuropsychological assessment may help to better understand which neurocognitive processes are affected by dissociation and how this may translate into altered affective-cognitive functioning. It has been proposed that dissociation specifically involves diminished recollection of trauma-related information [126], while the existing literature in the field is still heterogeneous [65]. To elucidate this relationship, future neuroimaging studies in BPD may investigate the effect of dissociation on processing (e.g., autobiographical recall) of trauma-related material compared to generally negative information. The combination of dissociation induction and affective-cognitive neuropsychological tasks in neuroimaging research may contribute to a better understanding of this relationship. Future studies may combine neuroimaging techniques with advanced methods of ecological momentary assessment in daily life to elucidate how specific dissociative symptoms (e.g., derealization versus dissociative amnesia) may relate to certain neural response patterns. A broader assessment of dissociative experiences in everyday life situations might also help to enhance our understanding of the ecological validity of experimental paradigms, which are used to experimentally induce dissociation, such as the script-driven imagery paradigm. More specifically, future studies may address how well naturally occurring dissociative symptoms correspond to dissociative symptoms provoked in the laboratory, e.g., by dissociation scripts.

Neuroimaging research on dissociation may also help to identify neural processes relevant for psychotherapy. Dissociative symptoms were found to hinder treatment outcome in BPD [16–19] and other psychiatric groups [127, 128], possibly by interfering with emotional learning and memory, e.g., habituation processes during exposure therapy [12–14, 96]. In a recent intervention study, a computerized (continuous) monitoring of dissociative states and suggestions for anti-dissociative skills had a beneficial effect on exposure exercises during trauma therapy [129]. Combining such interventions with repeated neuroimaging assessment (before and after treatment) could help to identify neural pathways associated with a reduction of dissociative symptoms [55]. Moreover, there is preliminary evidence that dissociative symptoms decrease along with a neurofeedback training targeted on amygdala activity in response to aversive images [95, 96], while it remains unclear which processes (e.g., enhanced top-down control versus bottom-up processes of emotion processing, such as increased emotional awareness, emotional clarity, and acceptance of emotions) may be involved. Neurofeedback training (real-time fMRI) may be a promising add-on intervention to help individuals gain more control over dissociative processes, i.e., reduce them at moments when these processes are disruptive and therefore maladaptive [130]. So far, very little is known about neural mechanisms that may be key modulators in this relationship and more studies are needed to identify brain areas that may be possible targets for such neurofeedback interventions.

#### Conclusion

Dissociative symptoms appear to have a significant impact on affective-cognitive functioning in BPD and should be taken into account in future research and therapy, even when not being the major focus. While the precise neural mechanisms of dissociation remain elusive, there is evidence for reduced activity in limbic(-related) temporal areas (amygdala, superior temporal gyrus, fusiform gyrus), increased frontal activity (inferior frontal gyrus, dlPFC), and altered interactions between these regions. More research with larger sample sizes and clinical control groups, combining different neuroimaging techniques (e.g., resting-state and task-related fMRI) with clinical measures and behavioral tasks, is needed to improve the understanding of dissociation in BPD.

## References

[1] Chopra HD, Beatson JA (1986). Psychotic symptoms in borderline personality disorder. Am J Psychiatr.

[2] Jaeger SS, T., Uhlmann C, Flammer E, Bichescu-Burian D, Tschöke S (2017). Dissociation in patients with borderline personality disorder in acute inpatient care—a latent profile analysis. Compr Psychiatry.

[3] Korzekwa MI, Dell PF, Links PS, Thabane L, Fougere P (2009). Dissociation in borderline personality disorder: a detailed look. J Trauma Dissociation : Off J Int Soc Study Dissociation.

[4] Scalabrini A, Cavicchioli M, Fossati A, Maffei C (2017). The extent of dissociation in borderline personality disorder: a meta-analytic review. J Trauma Dissociation.

[5] Simeon D, Nelson D, Elias R, Greenberg J, Hollander E (2003). Relationship of personality to dissociation and childhood trauma in borderline personality disorder. CNS Spectrums.

[6] Zanarini MC (2000). Childhood experiences associated with the development of borderline personality disorder. Psychiatr Clin N Am.

[7] American Psychiatric Association, APA, *Diagnostic and statistical manual of mental disorders, 5th ed*. 5th ed. 2013: American Psychiatric Association.

[8] Banich MT, Mackiewicz KL, Depue BE, Whitmer AJ, Miller GA, Heller W (2009). Cognitive control mechanisms, emotion and memory: a neural perspective with implications for psychopathology. Neurosci Biobehav Rev.

[9] Ford JD, Courtois CA (2014). Complex PTSD, affect dysregulation, and borderline personality disorder. Borderline Personal Disord Emot Dysregul.

[10] Laddis A, Dell PF, Korzekwa M (2017). Comparing the symptoms and mechanisms of “dissociation” in dissociative identity disorder and borderline personality disorder. J Trauma Dissociation.

[11] Stiglmayr CE, Ebner-Priemer UW, Bretz J, Behm R, Mohse M, Lammers CH, Anghelescu IG, Schmahl C, Schlotz W, Kleindienst N, Bohus M (2008). Dissociative symptoms are positively related to stress in borderline personality disorder. Acta Psychiatr Scand.

[12] Ebner-Priemer UW (2009). Emotional learning during dissociative states in borderline personality disorder. J Psychiatry Neurocience.

[13] Haaland VO, Landro NI (2009). Pathological dissociation and neuropsychological functioning in borderline personality disorder. Acta Psychiatr Scand.

[14] Löffler-Stastka H, Szerencsics M, Blüml V (2009). Dissociation, trauma, affect regulation and personality in patients with a borderline personality organization. Bull Menn Clin.

[15] Paret C, Hoesterey S, Kleindienst N, Schmahl C (2016). Associations of emotional arousal, dissociation and symptom severity with operant conditioning in borderline personality disorder. Psychiatry Res.

[16] Arntz A, Stupar-Rutenfrans S, Bloo J, van Dyck R, Spinhoven P (2015). Prediction of treatment discontinuation and recovery from borderline personality disorder: results from an RCT comparing schema therapy and transference focused psychotherapy. Behav Res Ther.

[17] Kleindienst N, Limberger MF, Ebner-Priemer UW, Keibel-Mauchnik J, Dyer A, Berger M, Schmahl C, Bohus M (2011). Dissociation predicts poor response to dialectial behavioral therapy in female patients with borderline personality disorder. J Personal Disord.

[18] Kleindienst N, Priebe K, Görg N, Dyer A, Steil R, Lyssenko L, Winter D, Schmahl C, Bohus M (2016). State dissociation moderates response to dialectical behavior therapy for posttraumatic stress disorder in women with and without borderline personality disorder. Eur J Psychotraumatol.

[19] Spitzer C, Barnow S, Freyberger HJ, Joergen Grabe H (2007). Dissociation predicts symptom-related treatment outcome in short-term inpatient psychotherapy. Aust N Z J Psychiatry.

[20] Krause-Utz A, Frost R, Winter D, Elzinga BM (2017). Dissociation and alterations in brain function and structure: implications for borderline personality disorder. Current Psychiatry Reports.

[21] Krystal, J.H., et al., *Recent developments in the neurobiology of dissociation. Implications for posttraumatic stress disorder*, in *Handbook of Dissociation. Theoretical, Empirical, and Clinical Perspectives* L.K. Michelson and W.J. Ray, Editors. 1996, Plenum Press: New York, NY p 163-190.

[22] Lanius RA, Vermetten E, Loewenstein RJ, Brand B, Schmahl C, Bremner JD, Spiegel D (2010). Emotion modulation in PTSD: clinical and neurobiological evidence for a dissociative subtype. Am J Psychiatr.

[23] Lanius RA, Brand B, Vermetten E, Frewen PA, Spiegel D (2012). The dissociative subtype of posttraumatic stress disorder: rationale, clinical and neurobiological evidence, and implications. Depress Anxiety.

[24] Spiegel D, Loewenstein RJ, Lewis-Fernández R, Sar V, Simeon D, Vermetten E, Cardeña E, Brown RJ, Dell PF (2011). Dissociative disorders in DSM-5. Depress Anxiety.

[25] Vermetten E, Spiegel D (2014). Trauma and dissociation: implications for borderline personality disorder. Curr Psychiatry Rep.

[26] Spiegel D, Cardena E (1991). Disintegrated experience: the dissociative disorders revisited. J Abnorm Psychol.

[27] Holmes EA (2005). Are there two qualitatively distinct forms of dissociation? A review and some clinical implications. Clin Psychol Rev.

[28] Waller NG, Putnam FW, Bernstein Carlson E (1996). Types of dissociation and dissociative types: a taxometric analysis of dissociative experiences. Psychol Methods.

[29] Brand BL, Lanius RA (2014). Chronic complex dissociative disorders and borderline personality disorder: disorders of emotion dysregulation?. Borderline Personal Disord Emot Dysregul.

[30] Sar V, Alioğlu F, Akyuz G (2017). Depersonalization and derealization in self-report and clinical interview: the spectrum of borderline personality disorder, dissociative disorders, and healthy controls. J Trauma Dissociation.

[31] Sar, V., Alioğlu, F., Akyuz, G., Tayakısı, E., Öğülmüş, E. F., Sönmez, D., *Awareness of identity alteration and diagnostic preference between borderline personality disorder and dissociative disorders.* J Trauma Dissociation, [Epub ahead of print] **2016 Dec 5**: p. 1–17. 10.1080/15299732.2016.1267684 27918876

[32] Elbert, T., et al., *The influence of organized violence and terror on brain and mind—a co-constructive perspective*, in *Lifespan development and the brain: the perspective of biocultural co-constructivism*, P. Baltes, P. Reuter-Lorenz, and F. Rösler, Editors. 2006, University Press: Cambridge, UK p 326-349.

[33] Gershuny BS, Thayer JF (1999). Relations among psychological trauma, dissociative phenomena, and trauma-related distress: a review and integration. Clin Psychol Rev.

[34] Marmar, C.R., D.S. Weiss, and T.J. Metzler, Peritraumatic dissociation and posttraumatic stress disorder, in *trauma, memory and dissociation*, J.D. Bremner and C.R. Marmar, Editors. 1998, American Psychiatric Press, Inc.: Washington, DC p 229-247.

[35] Nijenhuis ERS, Spinhoven P, van Dyck R, van der Hart O, Vanderlinden J (1998). Degree of somatoform and psychological dissociation in dissociative disorder is correlated with reported trauma. J Trauma Stress.

[36] Roelofs K, Hoogduin KA, Keijsers GP, Näring GW, Moene FC, Sandijck P (2002). Hypnotic susceptibility in patients with conversion disorder. J Abnorm Psychol.

[37] Roelofs K, Spinhoven P, Sandijck P, Moene FC, Hoogduin KA (2005). The impact of early trauma and recent life-events on symptom severity in patients with conversion disorder. J Nerv Ment Dis.

[38] Schauer M, Elbert T (2010). Dissociation following traumatic stress: etiology and treatment. J Psychol.

[39] Shearer SL (1994). Dissociative phenomena in women with borderline personality disorder. Am J Psychiatr.

[40] Spinhoven P, Roelofs K, Moene F, Kuyk J, Nijenhuis E, Hoogduin K, Van Dyck R (2004). Trauma and dissociation in conversion disorder and chronic pelvic pain. Int J Psychiatry Med.

[41] Van Den Bosch LM (2003). Trauma, dissociation, and posttraumatic stress disorder in female borderline patients with and without substance abuse problems. Aust N Z J Psychiatry.

[42] van der Hart O (2004). Trauma-related dissociation: conceptual clarity lost and found. Aust N Z J Psychiatry.

[43] Van der Kolk, B.A., McFarlane A.C., and L. Weisaeth, *Traumatic stress: the effects of overwhelming experience on mind, body, and society*. 1996, New York, NY: The Guilford Press.

[44] van der Kolk BA, van der Hart O (1989). Pierre Janet and the breakdown of adaptation in psychological trauma. Am J Psychiatr.

[45] Dutra L, Bureau JF, Holmes B, Lyubchik A, Lyons-Ruth K (2009). Quality of early care and childhood trauma: a prospective study of developmental pathways to dissociation. J Nerv Ment Dis.

[46] Ogawa JR, Sroufe LA, Weinfield NS, Carlson EA, Egeland B (1997). Development and the fragmented self: longitudinal study of dissociative symptomatology in a nonclinical sample. Dev Psychopathol.

[47] Cannon WB (1929). Bodily changes in pain, hunger, fear, and range.

[48] Fanselow, M.S. and L.S. Lester, *A functional behavioristic approach to aversively modtivated behavior: predatory immenence as a determinant of the topography of the defensive behavior*, in *Evolution and learning*, R.C. Bolles and M.D. Breecher, Editors. 1988, Erlbaum: Hilsdale, NJ p 185-212.

[49] Hagenaars MA, Oitzl M, Roelofs K (2014). Updating freeze: aligning animal and human research. Neurosci Biobehav Rev.

[50] Nesse RM (1999). Proximate and evolutionary studies of anxiety, stress and depression: synergy at the interface. Neurosci Biobehav Rev.

[51] Van der Kolk, B.A., O. Van der Hart, and C.R. Marmar, *Dissociation and information processing in posttraumatic stress disorder*, in *Traumatic Stress*, B.A. Van der Kolk, A.C. McFarlane, and L. Weisaeth, Editors. 1996, Guilford: New York, NY. p. 303-330.

[52] Frewen PA, Lanius RA (2006). Neurobiology of dissociation: unity and disunity in mind-body-brain. Psychiatr Clin North Am.

[53] Bennett DC, Modrowski CA, Kerig PK, Chaplo SD (2015). Investigating the dissociative subtype of posttraumatic stress disorder in a sample of traumatized detained youth. Psychol Trauma.

[54] van der Hart O, van Ochten JM, van Son MJM, Steele K, Lensvelt-Mulders G (2008). Relations among peritraumatic dissociation and posttraumatic stress: a critical review. J Trauma Dissociation.

[55] Lanius RA (2015). Trauma-related dissociation and altered states of consciousness: a call for clinical, treatment, and neuroscience research. Eur J Psychotraumatol.

[56] Conway MA, Pleydell-Pearce CW (2000). The construction of autobiographical memories in the self-memory system. Psychol Rev.

[57] Bremner, J.D., et al., *Trauma, memory, and dissociation: an integrative formulation.* Am Psychiatric Assoc Press, 1998: p. 365–402.

[58] Brewin CR (2001). A cognitive neuroscience account of posttraumatic stress disorder and its treatment. Behav Res Ther.

[59] Brewin CR, Dalgleish T, Joseph S (1996). A dual representation theory of posttraumatic stress disorder. Psychol Rev.

[60] Ehlers A, Clark DM (2000). A cognitive model of posttraumatic stress disorder. Behav Res Ther.

[61] Lensvelt-Mulders G, van der Hart O, van Ochten JM, van Son MJM, Steele K, Breeman L (2008). Relations among peritraumatic dissociation and posttraumatic stress: a meta-analysis. Clin Psychol Rev.

[62] van der Velden PG, Wittmann L (2008). The independent predictive value of peritraumatic dissociation for PTSD symptomatology after type I trauma: a systematic review of prospective studies. Clin Psychol Rev.

[63] Bremner JD (2006). Traumatic stress: effects on the brain. Dialogues Clin Neurosci.

[64] Chiu CD, Yeh YY, Huang YM, Wu YC, Chiu YC (2009). The set switching function of nonclinical dissociators under negative emotion. J Abnorm Psychol.

[65] de Ruiter MB, Phaf RH, Elzinga BM, van Dyck R (2004). Dissociative style and individual differences in verbal working memory span. Conscious Cogn.

[66] Elzinga BM (2007). Neural correlates of enhanced working-memory performance in dissociative disorder: a functional MRI study. Psychol Med.

[67] Sierra M, Berrios GE (1998). Depersonalization: neurobiological perspectives. Biol Psychiatry.

[68] Phillips ML, Medford N, Senior C, Bullmore ET, Suckling J, Brammer MJ, Andrew C, Sierra M, Williams SCR, David AS (2001). Depersonalization disorder: thinking without feeling. Psychiatry Res.

[69] Phillips ML, Sierra M (2003). Depersonalization disorder: a functional neuroanatomical perspective. Stress.

[70] Sierra M, Senior C, Dalton J, McDonough M, Bond A, Phillips ML, O’Dwyer AM, David AS (2002). Autonomic response in depersonalization disorder. Arch Gen Psychiatry.

[71] Davis M, Whalen PJ (2001). The amygdala: vigilance and emotion. Mol Psychiatry.

[72] Ochsner KN, Gross JJ (2007). The neural architecture of emotion regulation. *The Handbook of Emotion Regulation*, J.J. Gross and R. Buck.

[73] Ketay S, Hamilton HK, Haas BW, Simeon D (2014). Face processing in depersonalization: an fMRI study of the unfamiliar self. Psychiatry Res.

[74] Lemche E, Sierra-Siegert M, David AS, Phillips ML, Gasston D, Williams SCR, Giampietro VP (2016). Cognitive load and autonomic response patterns under negative priming demand in depersonalization-derealization disorder. Eur J Neurosci.

[75] Lemche E, Brammer MJ, David AS, Surguladze SA, Phillips ML, Sierra M, Williams SCR, Giampietro VP (2013). Interoceptive-reflective regions differentiate alexithymia traits in depersonalization disorder. Psychiatry Res.

[76] Greicius M (2008). Resting-state functional connectivity in neuropsychiatric disorders. Curr Opin Neurol.

[77] Greicius MD, Krasnow B, Reiss AL, Menon V (2003). Functional connectivity in the resting brain: a network analysis of the default mode hypothesis. Proc Natl Acad Sci U S A.

[78] Raichle ME, MacLeod AM, Snyder AZ, Powers WJ, Gusnard DA, Shulman GL (2001). A default mode of brain function. Proc Natl Acad Sci U S A.

[79] Buckner, R.L., Andrews-Hanna, J. R., Schacter, D. L., *The brain’s default network: anatomy, function, and relevance to disease.* Annals of the New York Academy of Science, 2008. 1124: p. 1–38. 10.1196/annals.1440.011 18400922

[80] Buckner, R.L., Vincent, J. L. , Unrest at rest: default activity and spontaneous network correlations*.* NeuroImage, 2007. 37(34): p. 1091–1096; discussion 1097-1099, 10.1016/j.neuroimage.2007.01.010. 10.1016/j.neuroimage.2007.01.010 17368915

[81] Laird AR, Eickhoff SB, Li K, Robin DA, Glahn DC, Fox PT (2009). Investigating the functional heterogeneity of the default mode network using coordinate-based meta-analytic modeling. J Neurosci.

[82] Simeon D, Guralnik O, Hazlett EA, Spiegel-Cohen J, Hollander E, Buchsbaum MS (2000). Feeling unreal: a PET study of depersonalization disorder. Am J Psychiatr.

[83] Sedeno L (2014). How do you feel when you can't feel your body? Interoception, functional connectivity and emotional processing in depersonalization-derealization disorder. PLoS One.

[84] Critchley HD, Mathias CJ, Dolan RJ (2001). Neural activity in the human brain relating to uncertainty and arousal during anticipation. Neuron.

[85] Dosenbach NU (2006). A core system for the implementation of task sets. Neuron.

[86] Menon V, Uddin LQ (2010). Saliency, switching, attention and control: a network model of insula function. Brain Struct Funct.

[87] Lanius RA, Williamson PC, Boksman K, Densmore M, Gupta M, Neufeld RWJ, Gati JS, Menon RS (2002). Brain activation during script-driven imagery induced dissociative responses in PTSD: a functional magnetic resonance imaging investigation. Biol Psychiatry.

[88] Lanius RA, Williamson PC, Bluhm RL, Densmore M, Boksman K, Neufeld RWJ, Gati JS, Menon RS (2005). Functional connectivity of dissociative responses in posttraumatic stress disorder: a functional magnetic resonance imaging investigation. Biol Psychiatry.

[89] Krystal, J.H., et al., *Toward a cognitive neuroscience of dissociation and altered memory functions in post-traumatic stress disorder*, M.J. Friedman, D.S. Charney, and A.Y. Deutch, Editors. 1995, Lippincott-Raven Publishers: Philadelphia. p. 228–269.

[90] Krystal, J.H., et al., *The emerging neurobiology of dissociation: implications for treatment of posttraumatic stress disorder*, J.D. Bremner and C.R. Marmar, Editors. 1998, American Psychiatric Press: Washington DC. p. 321–363.

[91] Felmingham K, Kemp AH, Williams L, Falconer E, Olivieri G, Peduto A, Bryant R (2008). Dissociative responses to conscious and non-conscious fear impact underlying brain function in post-traumatic stress disorder. Psychol Med.

[92] Moser DA, Aue T, Wang Z, Rusconi Serpa S, Favez N, Peterson BS, Schechter DS (2013). Limbic brain responses in mothers with post-traumatic stress disorder and comorbid dissociation to video clips of their children. Stress.

[93] Nicholson AA, Densmore M, Frewen PA, Théberge J, Neufeld RWJ, McKinnon MC, Lanius RA (2015). The dissociative subtype of posttraumatic stress disorder: unique resting-state functional connectivity of basolateral and centromedial amygdala complexes. Neuropsychopharmacology.

[94] Nicholson AA, Friston KJ, Zeidman P, Harricharan S, McKinnon MC, Densmore M, Neufeld RWJ, Théberge J, Corrigan F, Jetly R, Spiegel D, Lanius RA (2017). Dynamic causal modeling in PTSD and its dissociative subtype: bottom-up versus top-down processing within fear and emotion regulation circuitry. Hum Brain Mapp.

[95] Nicholson AA, Rabellino D, Densmore M, Frewen PA, Paret C, Kluetsch R, Schmahl C, Théberge J, Neufeld RW, McKinnon MC, Reiss J, Jetly R, Lanius RA (2017). The neurobiology of emotion regulation in posttraumatic stress disorder: amygdala downregulation via real-time fMRI neurofeedback. Hum Brain Mapp.

[96] Paret C, Kluetsch R, Zaehringer J, Ruf M, Demirakca T, Bohus M, Ende G, Schmahl C (2016). Alterations of amygdala-prefrontal connectivity with real-time fMRI neurofeedback in BPD patients. Soc Cogn Affect Neurosci.

[97] Harricharan S, Rabellino D, Frewen PA, Densmore M, Théberge J, McKinnon MC, Schore AN, Lanius RA (2016). fMRI functional connectivity of the periaqueductal gray in PTSD and its dissociative subtype. Brain and Behavior.

[98] Tursich M, Ros T, Frewen PA, Kluetsch RC, Calhoun VD, Lanius RA (2015). Distinct intrinsic network connectivity patterns of post-traumatic stress disorder symptom clusters. Acta Psychiatr Scand.

[99] Reinders AA (2006). Psychobiological characteristics of dissociative identity disorder: a symptom provocation study. Biol Psychiatry.

[100] Reinders AA (2014). Opposite brain emotion-regulation patterns in identity states of dissociative identity disorder: a PET study and neurobiological model. Psychiatry Res.

[101] Irle E, Lange C, Sachsse U (2005). Reduced size and abnormal asymmetry of parietal cortex in women with borderline personality disorder. Biol Psychiatry.

[102] Rusch N (2007). Inferior frontal white matter microstructure and patterns of psychopathology in women with borderline personality disorder and comorbid attention-deficit hyperactivity disorder. NeuroImage.

[103] Niedtfeld I (2013). Voxel-based morphometry in women with borderline personality disorder with and without comorbid posttraumatic stress disorder. PLoS One.

[104] Lange C, Kracht L, Herholz K, Sachsse U, Irle E (2005). Reduced glucose metabolism in temporo-parietal cortices of women with borderline personality disorder. Psychiatry Res.

[105] Sar V, Unal SN, Ozturk E (2007). Frontal and occipital perfusion changes in dissociative identity disorder. Psychiatry Res.

[106] Wolf RC, Thomann PA, Sambataro F, Vasic N, Schmid M, Wolf ND (2012). Orbitofrontal cortex and impulsivity in borderline personality disorder: an MRI study of baseline brain perfusion. Eur Arch Psychiatry Clin Neurosci.

[107] Wolf RC (2011). Aberrant connectivity of resting-state networks in borderline personality disorder. Journal of psychiatry & neuroscience: JPN.

[108] Krause-Utz A, et al. Amygdala and anterior cingulate resting-state functional connectivity in borderline personality disorder patients with a history of interpersonal trauma. Psychol Med. 2014:1–13. 10.1017/S0033291714000324 25066544

[109] Bernstein EM, Putnam FW (1986). Development, reliability, and validity of a dissociation scale. J Nerv Ment Dis.

[110] Hazlett EA, Zhang J, New AS, Zelmanova Y, Goldstein KE, Haznedar MM, Meyerson D, Goodman M, Siever LJ, Chu KW (2012). Potentiated amygdala response to repeated emotional pictures in borderline personality disorder. Biol Psychiatry.

[111] Krause-Utz A, Oei NYL, Niedtfeld I, Bohus M, Spinhoven P, Schmahl C, Elzinga BM (2012). Influence of emotional distraction on working memory performance in borderline personality disorder. Psychol Med.

[112] Krause-Utz A (2014). Amygdala and dorsal anterior cingulate connectivity during an emotional working memory task in borderline personality disorder patients with interpersonal trauma history. Front Hum Neurosci.

[113] Kluetsch RC, Schmahl C, Niedtfeld I, Densmore M, Calhoun VD, Daniels J, Kraus A, Ludaescher P, Bohus M, Lanius RA (2012). Alterations in default mode network connectivity during pain processing in borderline personality disorder. Arch Gen Psychiatry.

[114] Kraus A, Esposito F, Seifritz E, di Salle F, Ruf M, Valerius G, Ludaescher P, Bohus M, Schmahl C (2009). Amygdala deactivation as a neural correlate of pain processing in patients with borderline personality disorder and co-occurrent posttraumatic stress disorder. Biol Psychiatry.

[115] Krause-Utz A, et al. Classical conditioning in borderline personality disorder: an fMRI study. Eur Arch Psychiatry Clin Neurosci. 2015; 10.1007/s00406-015-0593-1 25814470

[116] Wingenfeld K, Rullkoetter N, Mensebach C, Beblo T, Mertens M, Kreisel S, Toepper M, Driessen M, Woermann FG (2009). Neural correlates of the individual emotional Stroop in borderline personality disorder. Psychoneuroendocrinology.

[117] Winter D, Krause-Utz A, Lis S, Chiu CD, Lanius RA, Schriner F, Bohus M, Schmahl C (2015). Dissociation in borderline personality disorder: disturbed cognitive and emotional inhibition and its neural correlates. Psychiatry Res.

[118] Krause-Utz, A., Winter, D., Schriner, F., Chiu, C. D., Lis, S., Spinhoven, P., Bohus, M., Schmahl, C., Elzinga, B. M., *Reduced amygdala reactivity and impaired working memory during dissociation in borderline personality disorder.* European Archives of psychiatry and Clin Neurosci, 2017 [Epub ahead of print]. 10.1007/s00406-017-0806-x PMC5956011 28526931

[119] Ludascher P (2010). Pain sensitivity and neural processing during dissociative states in patients with borderline personality disorder with and without comorbid posttraumatic stress disorder: a pilot study. J Psychiatry Neurosci : JPN.

[120] Aron AR, Robbins TW, Poldrack RA (2014). Inhibition and the right inferior frontal cortex: one decade on. Trends Cogn Sci.

[121] Ludascher P (2007). Elevated pain thresholds correlate with dissociation and aversive arousal in patients with borderline personality disorder. Psychiatry Res.

[122] Krause-Utz A, Winter D, Niedtfeld I, Schmahl C (2014). The latest neuroimaging findings in borderline personality disorder. Curr Psychiatry Rep.

[123] Schulze L, Schmahl C, Niedtfeld I (2016). Neural correlates of disturbed emotion processing in borderline personality disorder: a multimodal meta-analysis. Biol Psychiatry.

[124] Krause-Utz A, Schmahl C (2016). A more global look at altered neural structure and resting-state function in borderline personality disorder. Biol Psychiatry.

[125] Nichols TE, Das S, Eickhoff SB, Evans AC, Glatard T, Hanke M, Kriegeskorte N, Milham MP, Poldrack RA, Poline JB, Proal E, Thirion B, Van Essen DC, White T, Yeo BT (2017). Best practices in data analysis and sharing in neuroimaging using MRI. Nat Neurosci.

[126] DePrince AP, Freyd JJ (2004). Forgetting trauma stimuli. Psychol Sci.

[127] Michelson L, June K, Vives A, Testa S, Marchione N (1998). The role of trauma and dissociation in cognitive-behavioral psychotherapy outcome and maintenance for panic disorder with agoraphobia. Behaviour Research & Therapy.

[128] Rufer M, Held D, Cremer J, Fricke S, Moritz S, Peter H, Hand I (2006). Dissociation as a predictor of cognitive behavior therapy outcome in patients with obsessive-compulsive disorder. Psychother Psychosom.

[129] Görg, N., Priebe, K., Deuschel, T., Schüller, M., Schriner, F., Kleindienst, N., Ludäscher, P., Schmahl, C., Bohus, M. , *Computer-assisted in sensu exposure for posttraumatic stress disorder: development and evaluation.* JMIR Ment Health, 2016. **3**(2): p. e27. . 10.2196/mental.5697 PMC4936796 27277899

[130] Lieb K, Zanarini MC, Schmahl C, Linehan MM, Bohus M (2004). Borderline personality disorder. Lancet.

